# Epitome of Mental Diseases

**Published:** 1893-06

**Authors:** 


*Epitome of Mental Diseases.* By JAMES SHAW, M.D. Pp. xv., 345. Bristol: JOHN WRIGHT & CO. 1892.

The title of this book to some extent disarms criticism. An epitome is hardly to be judged on the same lines as an ordinary text-book. Still, as the author in his preface claims a use for his book as an introduction for students to the more comprehensive treatises and exhaustive monographs, it is necessary to examine this claim a little closely.

Although not without precedents in its favour, we confess we do not like the arrangement whereby the consideration of a disease is split up, under the headings of Etiology, Symptoms, Prognosis, Pathology, and Treatment, into sections widely separated from each other. The book contains an enormous quantity of information, and is a monument of research and industry. But we are bound to say that clearness is often sacrificed to compression, and that the student who has no previous knowledge to guide him will be tempted to lay it down, hopelessly bewildered and confused. To him the very special and technical terminology often adopted will be in itself a stumbling-block, and the various classifications, starting from different bases, more than a little perplexing. For such a reader, the book is wholly unsuited. On the other hand, it will prove valuable to the expert physician, who is able to simplify and illustrate its statements by the cases he can recall in his own practice, while his experience will also enable him to make a decision on various contentious and conflicting matters and opinions, which Dr. Shaw plainly sets out in the text, but still sometimes leaves in doubt. To the teacher and lecturer, who desires a handy and full synopsis of the ideas and practice of various authors on psychology, the work will prove of special service.

Apart from its method and manner of presentation, little fault is to be found with its matter. The chapter on Pathology is especially worth study, and fully describes the experiments and clinical observations bearing on cerebral localisation. The book altogether has been carefully compiled, and is generally trustworthy. We are, however, somewhat surprised in so recent a work as this to see no mention of post-influenzal insanity. We strongly protest against there being any justification for the removal of portions of the cortex with a sharp spoon, for the relief of that train of symptoms it is now fashionable to call paranoia. And while the section on Prognosis is good, Voisin's experience that cases of general paralysis recover under cold baths is most exceptional. When the diagnosis in this disease is undoubted, permanent recovery under any form of medication is so very rare as hardly to justify its expectation as a possibility.

